# Creating a Pediatric Prehospital Destination Decision Tool Using a Modified Delphi Method

**DOI:** 10.3390/children8080658

**Published:** 2021-07-29

**Authors:** Jennifer F. Anders, Jennifer N. Fishe, Kyle A. Fratta, Jessica H. Katznelson, Matthew J. Levy, Richard Lichenstein, Michael G. Milin, Joelle N. Simpson, Theresa A. Walls, Heather L. Winger

**Affiliations:** 1 Department of Pediatrics, Johns Hopkins University, Baltimore, MD 21287, USA; frattaka@upmc.edu (K.A.F.); Jkatzne1@jhmi.edu (J.H.K.); 2 Department of Emergency Medicine, University of Florida–Jacksonville, Jacksonville, FL 32224, USA; Jennifer.fishe@jax.ufl.edu; 3 Department of Emergency Medicine, University of Pittsburgh Medical Center-Harrisburg, Harrisburg, PA 15213, USA; 4 Department of Emergency Medicine, Johns Hopkins University, Baltimore, MD 21287, USA; levy@jhmi.edu (M.J.L.); michaelgmillin@gmail.com (M.G.M.); 5 Division of Pediatric Emergency Medicine, University of Maryland School of Medicine, Baltimore, MD 21201, USA; rlichenstein@som.umaryland.edu; 6 Department of Emergency Medicine, Children’s National Hospital, Washington, DC 20010, USA; jnsimpso@childrensnational.org; 7 Division of Emergency Medicine, Children’s Hospital of Philadelphia, Philadelphia, PA 19104, USA; wallst1@email.chop.edu; 8 Baltimore County Fire Department, Towson, MD 21286, USA; hlalexander380@gmail.com

**Keywords:** emergency medical services (EMS), prehospital, pediatrics, emergency care, triage, transport, regionalization, specialty care

## Abstract

Decisions for patient transport by emergency medical services (EMS) are individualized; while established guidelines help direct adult patients to specialty hospitals, no such pediatric equivalents are in wide use. When children are transported to a hospital that cannot provide definitive care, care is delayed and may cause adverse events. Therefore, we created a novel evidence-based decision tool to support EMS destination choice. A multidisciplinary expert panel (EP) of stakeholders reviewed published literature. Four facility capability levels for pediatric care were defined. Using a modified Delphi method, the EP matched specific conditions to a facility pediatric-capability level in a draft tool. The literature review and EP recommendations identified seventeen pediatric medical conditions at risk for secondary transport. In the first voting round, two were rejected, nine met consensus for a specific facility capability level, and six did not reach consensus on the destination facility level. A second round reached consensus on a facility level for the six conditions as well as revision of one previously rejected condition. In the third round, the panel selected a visual display format. Finally, the panel unanimously approved the PDTree. Using a modified Delphi technique, we developed the PDTree EMS destination decision tool by incorporating existing evidence and the expertise of a multidisciplinary panel.

## 1. Introduction

More than one million pediatric patients are transported by prehospital emergency medical services (EMS) in the United States each year [1]. Following initial transport to an emergency department (ED), some pediatric patients then require secondary interfacility transport to specialty hospitals for definitive care [2]. Due to the wide variability in the pediatric capabilities of hospitals and the increasing centralization of pediatric care with the reduction of community pediatric inpatient beds, rates of secondary transport have dramatically increased, including for straightforward pediatric emergencies [3,4,5,6].

Pediatric patients are particularly susceptible to negative consequences of secondary transport [7,8]. Reported consequences include transport-related adverse events, increased lengths of stay, increased morbidity and delays in care [7,8,9,10,11]. Although transferring hospitals typically initiate interfacility transport rapidly, the average pediatric interfacility transport takes three hours to complete [2,12,13]. Even after arrival to definitive care, secondary transported children may still be subject to deleterious effects of undertreatment at the initial site or duplicate testing and imaging at the second site [2,14,15].

Despite negative consequences from undertriage, most children transported by EMS do not need a high-capability pediatric facility. Overtriage increases travel burdens on families and represents an inefficient use of EMS resources, which could harm other patients who are also awaiting EMS care. Therefore, an ideal decision support tool would match pediatric patient needs with facility pediatric capability. For prehospital EMS providers, hospital destination choices are multifactorial, guided by patient condition, transport times, jurisdictional resources, local hospital capabilities, and patient/family preference. A statewide study from Florida found that one-third of pediatric transport decisions were due to patient/family preference [16]. Evidence-based guidelines (EBGs) have been developed to aid the EMS decision-making for adult patients suffering from trauma, myocardial infarction, stroke, psychiatric, and geriatric emergencies [17,18,19,20,21,22]. Those EBGs for direct transport protocols have demonstrated improved patient outcomes and EMS systems benefits [21,22,23,24]. While the all-ages trauma triage guidelines do address pediatric patients [17,25], rates of undertriage remain unacceptably high for injured children [26]. More significantly, no analogous guidelines exist for children with medical conditions.

To address that deficiency, the Pediatric Decision Tree (PDTree) was conceived as a pediatric prehospital destination decision support tool. The PDTree is designed to support EMS providers’ decision-making and guide them to transport pediatric patients to a facility capable of providing definitive care. Recognizing the multi-factorial transport decision processes required of EMS, creation of the PDTree proceeded in steps combining available evidence and multidisciplinary consensus.

## 2. Materials and Methods

### 2.1. Study Design

The modified Delphi technique was used to create the PDTree tool through four iterative rounds of voting that took place between March and May of 2017. The modified Delphi method guides a multidisciplinary group of subject matter experts to arrive at a consensus [27]. That methodology has previously been applied to healthcare settings, including for EMS decision tools [28,29,30,31]. In the modified Delphi technique, the research team presents questions or scenario-based cues to an expert panel (EP), and EP members vote on each question/cue independently [27]. The modified Delphi technique differs from the traditional Delphi method in that the questions are predetermined by the study team based on existing evidence [32]. The project considered EP members as research volunteers, and the study was approved by the Institutional Review Board of Johns Hopkins Medicine.

Thirty-four experts were invited, and twenty-two agreed to participate in the PDTree EP. A quorum of 12 voting members from five distinct stakeholder groups was defined to maintain a balance of stakeholders through all voting rounds: three emergency physicians (EM), three pediatric emergency physicians (PEM), two EMS physicians who serve as EMS agency medical directors, three EMS providers, and one patient/family representative. At each meeting of the EP, each of these 12 stakeholder positions was represented by one of the 22 EP members. EP members who were not designated as voting members for a specific meeting were invited to attend and contribute to the discussion.

### 2.2. Systematic Review of the Literature

To prepare for the modified Delphi process, three authors (KAF, JNF, JFA) performed a literature review utilizing multiple databases and the expertise of a medical librarian. MeSH terms are shown in Table 1. Search was limited to publications available in English, but no date or publication-type filters were used. Included for further review were 60 articles relevant to non-trauma pediatric secondary transport or prehospital direct transport guidance. Studies of conditions addressed by existing all-ages trauma triage guidelines were specifically excluded, as the novel tool is intended to supplement and not contradict existing guidance. Each article was independently reviewed and rated by two of the three authors. Ratings were based on the quality of evidence, relevance, and importance to prehospital destination choice. The authors met to discuss differences of opinion; disagreements were mediated by the third author. After consensus, 47 articles were included in the evidence review provided to EP members. A summary of that review was published previously [33].

Additionally, results from three preliminary studies designed to inform the PDTree’s development were presented to the EP. Those studies included a statewide assessment of interfacility transport patterns, a case-control study on conditions that resulted in secondary transport to a pediatric specialty center, and semi-structured interviews with EMS providers exploring current practice and attitudes toward pediatric transport destination choice [2,34].

Prior to the first meeting of the EP, members were provided with the literature review summary, copies of the 47 articles and summaries of the three preliminary studies. EP members were provided with a list of conditions identified by evidence review for potential inclusion on the tool and were asked to submit additional conditions for consideration.

### 2.3. First in-Person Meeting (Round 1)

Four levels of facility pediatric capability were created a priori—Closest Facility, Regional Pediatric Center, Comprehensive Pediatric Center, and Specialty/Trauma Pediatric Center. Those capability levels allow for hospital classification using publicly available information (Table 2).

A list of 17 conditions were presented to the panel members, and a timed discussion period was moderated by the study team. First, EP members considered if each condition warranted inclusion in the tool, and if so, to which facility capability level a pediatric patient with that condition should be transported (Table 3).

After 10 min, an electronic vote was held. Consensus was defined at 75% agreement for each question. If consensus was reached, the condition was either discarded or placed on the tool in the specified facility level. If consensus was not reached, an addition 10-min discussion period allowed EP members to suggest clarifications or specific subsets of the condition. One study team member moderated this discussion while another transcribed EP comments.

### 2.4. Item Specifications and Verbiage (Round 2)

The second round of EP voting sought to clarify included conditions that did not reach consensus on an optimal destination-facility capability level during the first round. During online voting, conditions were presented with alternate verbiage or additional specifications from the transcribed EP discussion along with the two facility capability levels that had the most votes in round 1. Consensus was defined as 60% agreement for a single specification/verbiage option and destination level.

Four draft tools were developed based on the consensuses from previous voting sessions. The drafts contained the same content but differed in organization and formatting. In a second in-person meeting, the EP voted for a preferred draft and discussed including EMS systems considerations such as transport time or distance limits (beyond which EMS units should divert to a closer hospital), use of helicopter-EMS (HEMS) services, and online medical direction. Those considerations were incorporated into the final draft of the PDTree tool, which was presented electronically to the EP for voting.

## 3. Results

In the first voting round, 9 of the 17 conditions reached consensus for optimal pediatric-facility capability level. The panel rejected two conditions, ”respiratory distress with an oxygen requirement” and “hypoxia”, for lack of clarity. Six conditions had consensus for inclusion but did not reach consensus for destination-facility capability level. The EP consensus for each condition by voting round is detailed in Table 3.

In the second round of voting, alternate verbiage or additional specifications were made, and consensus was reached for 8 conditions (Table 3). The previously rejected respiratory distress condition was reworded as “respiratory distress with hypoxia or serious signs and symptoms”. The condition “non-traumatic altered mental status” was dichotomized by age to create two distinct conditions. Table 4 shows the original and consensus wordings for the conditions with altered verbiage or specifications.

Three conditions generated robust discussion: shock, respiratory distress with an oxygen requirement, and altered mental status (non-trauma). While EP members felt strongly that patients in shock should be transported to a high-capability facility, they were reluctant to define vital signs specifications. Instead, the EP endorsed the inclusion of the validated prehospital Pediatric Assessment Triangle (PAT) by incorporating “abnormal PAT finding” in the “shock” condition [35]. Similarly, the “respiratory distress with an oxygen requirement” condition was revised to “respiratory distress with hypoxia or serious signs and symptoms” to allow EMS clinical decision-making. As with the PAT, this revision is supported by literature predicting which children require a higher level of care [2,36,37]. It also allows EMS providers to utilize the PDTree without pulse oximetry, which is not yet universally available in EMS.

In the discussion of altered mental status (AMS), EP members created subsets of patients by age (older or younger than 2 years), and the presence or absence of a known seizure disorder. Patients with apparent post-ictal AMS were determined appropriate for transport to the closest facility. For children 2 and older without seizure, EP consensus destination was a regional facility. For children younger than 2 years, the consensus destination was a comprehensive center due to concerns for possible abusive head trauma and higher likelihood of need for ICU admission.

**Figure 1:**
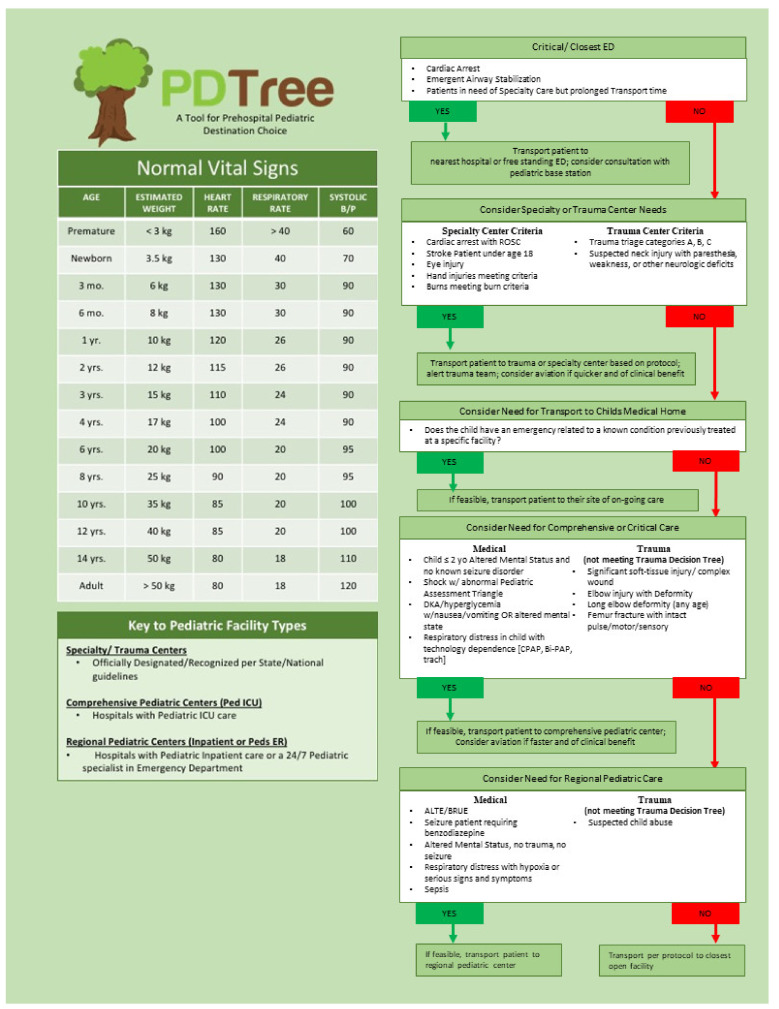
The Pediatric Decision Tree (PDTree) destination-decision support tool. Detailed criteria for Triage Categories and the Maryland Trauma Decision Tree referenced in the tool is found in [25] and are consistent with existing CDC trauma triage guidance [17]. Burn center criteria refer to the recommendations of the American Burn Association and can be found in [39].

At the second in-person meeting, the EP reviewed four draft tools. In a single vote, the EP reached consensus for the visual layout organizing conditions by destination-facility capability level in two columns (medical and trauma). Additionally, the EP discussed including recommendations for transport time or distance limits, helicopter-EMS (HEMS), and online medical direction. The EP voted for the tool to address those factors based on resource availability (e.g., “If feasible transport patient to … center”; “consider aviation if faster or of clinical benefit”—Figure 1), but to leave specific parameters to the discretion of local medical directors and jurisdictional leadership. The EP unanimously voted to include the medical home as a destination for emergencies related to established conditions. The EP unanimously voted against prompts for online medical direction in an effort to respect EMS provider and agency autonomy and avoid delays in care [38]. After this meeting, a final draft tool was generated and presented to the EP, which was unanimously approved in online voting. The completed PDTree tool is presented in Figure 1.

## 4. Discussion

The PDTree is an evidence-based decision support tool designed for EMS providers to choose an optimal destination for prehospital pediatric patients. The primary goal of the PDTree is to increase the proportion of children directly transported to a facility capable of definitive care, thereby reducing secondary transport, delays in care, and adverse events. However, that goal is balanced against overburdening EMS resources and operational capabilities.

In the absence of evidence-based guidance, EMS providers’ clinical discretion is used to determine the appropriate destination. That EMS subjective judgment is usually made on readily apparent clinical signs with an emphasis on the speed of decision-making [40]. Because of the emphasis on quick decisions and the difficulty in obtaining vital signs in pediatric patients, we developed a tool focused on clinical conditions and patient presentation rather than fixed physiologic criteria. Additionally, age-specific physiologic or vital signs used in isolation have been found to have only moderate predictive value in prehospital triage [41,42,43,44]. The EP chose to include age-based vital signs as a convenient reference on the tool (Figure 1).

In developing the PDTree, we explicitly defined pediatric-facility capability levels. However, definitions of pediatric-facility capability are not widely operative in EMS systems or hospital designation other than trauma/burn centers. The increasing centralization of pediatric resources and the opaque nature of true pediatric capabilities at individual hospitals poses a significant challenge for EMS providers to match pediatric patients’ perceived needs from their prehospital viewpoint to actual hospital capabilities [3,45,46]. In the United States, trauma-center designation is rigorously defined, guided by a governing body, and integrated into prehospital guidelines [17,46]. For non-trauma conditions, there are many barriers to creation of rigorous criteria for tiered pediatric-facility designation, but some states have successfully done so. Our experience developing the PDTree supports the calls by others that the continuum of pediatric emergency care would benefit from facility designations at both the ED and hospital level [45,46,47,48].

While individual EMS provider clinical decision-making will always be important, it is often inadequate in isolation and highly variable between providers [49]. Evidence-based direct transport protocols and tools have been implemented for a variety of adult clinical scenarios, with improved triage and patient outcomes. By incorporating EMS providers’ clinical findings within an evidence-based decision support tool, the PDTree aims to improve the efficiency of transporting more children to a destination capable of delivering definitive care.

An inherent concern with the modified Delphi technique is sustained and longitudinal expert participation. Because our EP members were busy professionals, we decided to empanel more than the minimum for quorum. We imposed a quorum for each stakeholder position to protect the relative position voting weight despite dropouts or inconsistent response rates between voting rounds. Another potential limitation to the modified Delphi technique is introduction of bias based on the cues provided to the EP members. We opted for non-presumptive language in forming our questions and allowed EP members to further define the question/cue for each condition.

Because the PDTree was developed by researchers and EP members from a single state, some elements may not be generalizable to other EMS systems. Maryland operates a statewide EMS system with shared protocols and operational resources, such as HEMS. Additionally, the three preliminary studies that were presented utilized Maryland data. Therefore, the PDTree may not directly translate for immediate operative use in other EMS systems. However, the PDTree was created with the intention that it could be adapted for use in diverse states and nations. The application of a PDTree tool to international systems may vary greatly with the scope of practice for EMS or the variable use of nurses or physicians in field response teams. However, the limited availability of pediatric specialty knowledge and care is universal. The open-ended language regarding use of HEMS or reasonable transport times/distances allows EMS systems to utilize the PDTree tool in the context of their operational resources. Additionally, diverse EMS systems may find it beneficial to utilize the process outlined in this paper to replicate consensus finding on optimal destination choice and adapt the PDTree’s evidence-based guidelines to their location.

The PDTree tool is expected to undergo revisions and adaptations in the future. Measurement of the impact of the tool includes patient health outcomes, system efficiency (reduction in secondary transport), and EMS operational resource costs. Expected revisions will address any concerns that arise in these measures. In addition, it is likely that future revisions will revise terminology or simplify language.

## 5. Conclusions

For decades, EMS providers have successfully utilized prehospital destination guidance for select high-risk adult conditions. The PDTree is presented here as a novel pediatric EMS destination decision tool. The modified Delphi technique allowed a multidisciplinary team of stakeholders to incorporate existing evidence with their own experience and reach consensus on the PDTree. The PDTree will undergo prospective testing to measure its impact on decision-making, patient outcomes, and EMS resource use.

## Figures and Tables

**Table 1:** Medical Subject Heading (MeSH) Terms used in PDTree Literature Review.

MeSH Terms Used in Literature Search, Grouped by Category
Adolescent Child Infant Pediatrics
Ambulance Emergency Medical Services Emergency Medical Technicians
Critical Illness Critical Care Intensive Care Unit Intensive Care Unit, Pediatric
Hospitalization Referral and Consultation Tertiary Care Centers
Patient Transfer Patient Transport Time-to-treatment Secondary Transport
Predictive Value of Tests Triage Vital Signs

**Table 2:** Definitions of Pediatric-Facility Capability Levels.

Hospital Classification	Pediatric Capabilities
Specialty Center	Trauma, burn, or other specialty center for pediatrics as certified by state or national governing body
Comprehensive Center	The presence of a Pediatric Intensive Care Unit and Pediatric OR/anesthesia services
Regional Center	The presence of a pediatric inpatient unit or pediatric physician present on site 24-h per day
Closest Facility	All other open facilities, including freestanding ED

**Table 3:** Conditions discussed by PDTree EP and voting results by rounds for inclusion on the PDTree and recommended destination-facility capability level.

#	Condition	Round 1 Consensus for Inclusion	Pediatric Facility Capability Level	Round 2 Consensus for Inclusion	Pediatric Facility Capability Level
1	Femur Fracture	Yes	Comprehensive		
2	Long Bone Fracture with Deformity	Yes	Comprehensive		
3	Suspected C-spine Injury	Yes	Trauma/Specialty Center		
4	Respiratory Distress with Oxygen Requirement	No	N/A	Yes-reworded	Regional
5	Respiratory Distress with Tracheostomy	Yes	Comprehensive		
6	Non-traumatic Altered Level of Consciousness	Yes	No Level of Care Determined	Yes-dichotomized by age	<2 years old-Comprehensive
					>2 years old-Regional
7	ALTE/BRUE	Yes	No Level of Care Determined	Yes	Regional
8	Sepsis High Risk	Yes	Comprehensive		
9	Sepsis Low Risk	Yes	Regional		
10	Complex Wound	Yes	No Level of Care Determined	Yes-reworded	Comprehensive
11	Eye Injury	Yes	Trauma/Specialty Center		
12	Children with Special Health Care Needs	Yes	Comprehensive		
13	Suspected Child Abuse	Yes	No Level of Care Determined	Yes	Regional
14	DKA/Hyperglycemia	Yes	Comprehensive		
15	Shock	Yes	No Level of Care Determined	Yes-Reworded	Comprehensive
16	Hypoxia	Yes	Do not include		
17	Seizure with Medication Administration by EMS	Yes	No Level of Care Determined	Yes-Reworded	Regional

**Table 4:** PDTree conditions that were reworded by the expert panel.

Original Wording (Round 1)	Revised Wording (Round 2)
Seizure Requiring Medication Administration by EMS	Seizure Requiring Benzodiazepine
Complex Wound	Significant soft-tissue injury/complex wound
Non-Trauma Altered Mental Status with no known seizure disorder	Dichotomized by age <2 yo and ≥2 yo
Shock	Shock with Abnormal Pediatric Assessment Triangle
Respiratory Distress with Oxygen Requirement	Respiratory Distress with Hypoxia or Serious Signs and Symptoms
